# Concomitant Traumatic Peroneal Tendon Dislocation and Medial Malleolus Fracture: A Case Report

**DOI:** 10.5704/MOJ.1803.014

**Published:** 2018-03

**Authors:** AR Vosoughi, MA Erfani

**Affiliations:** Bone and Joint Diseases Research Center, Shiraz University of Medical Sciences, Shiraz, Iran

**Keywords:** peroneal tendon subluxation, medial malleolus fracture, ankle, superior peroneal retinaculum

## Abstract

Peroneal tendon dislocation in association with medial malleolus fracture is a very rare traumatic injury to the ankle. A 19-year old male patient was referred after injury sustained in a motorcycle accident with car, with concomitant traumatic peroneal tendon dislocation and medial malleolus fracture. The possible mechanism of this unusual injury could have been sudden external rotation force to the pronated foot in full dorsiflexed position of the ankle. Diagnosis of peroneal tendon subluxation or dislocation should be carefully evaluated in patients with single medial malleolus fracture.

## Introduction

Peroneal tendon subluxation or dislocation is a rare traumatic injury to the ankle. Although, it was first described in a ballet dancer, other sport activities like skiing, soccer and basketball could result in traumatic dislocation of the peroneal tendon. Usually peroneal tendon subluxation or dislocation is mistaken or undiagnosed because of similarity of the mechanism of injury to that of lateral ankle sprain and lack of apparent findings in plain radiographs^1^.

Medial malleolus fracture usually occurs in association with fractures of lateral malleolus, posterior malleolus, proximal fibula, or with ligamentous injuries around the ankle joint. Concomitant traumatic peroneal tendon dislocation and medial malleolus fracture have been rarely reported in the literature^2,3^. We describe a case of minimally-displaced medial malleolus fracture in association with avulsion fracture of superior peroneal retinaculum to show the importance of small flake fractures around the ankle joint.

## Case Report

**Fig. 1:**
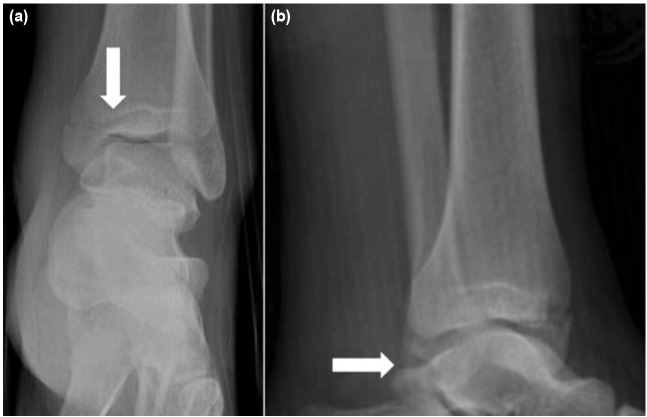
(a) Anteroposterior and (b) Lateral ankle radiographs of the patient on admission showed minimally-displaced fracture of medial malleolus and a suspicious avulsion fracture on posterior part of lateral malleolus.

**Fig. 2:**
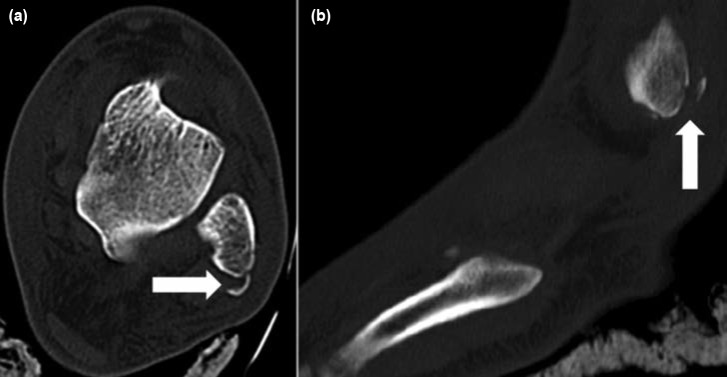
(a) Axial and (b) Sagittal CT scan show avulsion of superior peroneal retinaculum from the fibula.

A 19-year old man was referred with pain in left ankle after motor-vehicle accident. On physical examination, swelling and ecchymosis around the ankle joint especially on medial side was obvious. Significant tenderness on medial malleolus and little tenderness on lateral malleolus were noted on palpation of the ankle joint. Neurovascular function of the foot and ankle was normal. Radiographs disclosed minimally-displaced fracture of the medial malleolus and a barely visible small avulsion fracture on the posterodistal part of the lateral malleolus (Fig. 1). Computed tomography (CT) scan to determine the exact position of the avulsion fracture of lateral malleolus revealed a flake avulsion of superior peroneal retinaculum (SPR) from the posterior aspect of the lateral malleolus (Fig. 2).

After obtaining consent from the patient and under general anaesthesia in the operating room, open reduction and internal fixation of the medial malleolus fracture was carried out using a malleolar screw and a Kirschner wire was shortened and buried beneath the skin. Examination during surgery revealed dislocation of peroneal tendons to the anterior aspect of the lateral malleolus with passive full dorsiflexion of the ankle joint, following which, through an incision on the posterior aspect of the distal lateral malleolus, the avulsed superior peroneal retinaculum (SPR) was exposed. The flake of bone and SPR were anchored to the lateral malleolus with two sutures. Stability of the peroneal tendons was fully achieved.

**Fig. 3:**
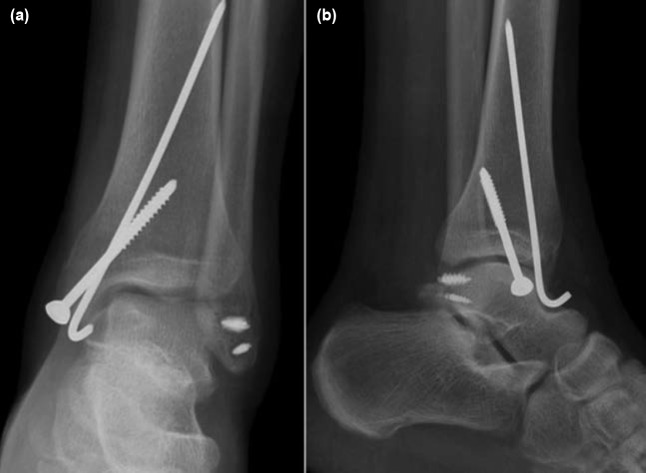
(a) Anteroposterior and (b) Lateral ankle radiographs of the patient six months after the surgery.

Postoperatively, non-weight bearing short leg cast was applied for six weeks followed by a short leg walking slab to initiate range of motion of ankle for four more weeks. At follow-up one year after surgery, the patient was completely satisfied with the healed fracture and tendon injury (Fig. 3) and had a full range of motion of the joint despite retained devices.

## Discussion

Diagnosis of flake fracture around the ankle joint is important because it usually shows avulsion of an essential ligament or an important retinaculum. Peroneal tendon subluxations have been classified by Eckert and Davis^4^. In Grade I, peroneal tendons slip anteriorly on intact fibrocartilaginous ridge of retrofibular groove. A Grade II subluxation of peroneal tendons is characterized by anterior slipping of tendons under elevated fibrocartilaginous ridge of retrofibular groove. In Grade III, avulsion fracture of SPR (the “fleck sign”) from fibula is seen. In the case presented here, the little bone fragment on the posterodistal aspect of fibula showed Grade III peroneal tendon dislocation. Grade III has the lowest incidence (13%) among all grades described by Eckert and Davis.

In order to explore the possible mechanism of this injury, we reviewed the literature. In our case and other reported cases of concomitant medial malleolus fracture and traumatic avulsed SPR^2,3^, the medial malleolus fracture was Type B or Type C by the Herscovici *et al* classification^5^. These types of medial malleolus fractures occurred with external rotation forces on the pronated foot by the Lauge-Hansen classification. It meant that none of the medial malleolus fractures in the reported cases resulted from shearing force as seen in supination-adduction injuries of the ankle.

Possible mechanism would be sudden external rotation force applied to the pronated foot in full dorsiflexed position of the ankle. When the ankle is in full dorsiflexion with contraction of the peroneal tendons in the active pronated position of the foot, the tendons are in direct forceful contact to the anterior part of SPR which is attached to the fibula. Sudden external rotation force in this position of the foot and ankle could initially tear the anterior inferior tibiofibular ligament and open the syndesmosis from anterior, as described in the second stage of pronation external rotation injury of Lauge-Hansen classification. These changes could finally rotate the fibula externally. Sudden forceful external rotation of the fibula against powerful contraction of peroneal tendons in the fully dorsiflexed ankle could cause avulsion of SPR from fibula (Grade III) or dislocation of peroneal tendons without avulsion fracture (Grade I or II). This theory could explain the mechanism of injury of concomitant traumatic peroneal tendons dislocation and medial malleolus fracture. This is only a theory and it should be tested in research on cadavers.

In conclusion, more attention should be paid to the lateral part of the ankle joint in patients with a solitary medial malleolus fracture of Type B or Type C by the Herscovici classification. When any small avulsed bone fragment from the posterior aspect of the lateral malleolus is revealed on radiography, instability of peroneal tendons must be looked for. After fixation of medial malleolus fracture under general anaesthesia, it is important to exclude any possible peroneal tendon subluxation even in the absence of evidence of bone fragment avulsion.

## Conflict of interest

The authors declare no conflicts of interest and no external funding was received in the preparation of this report.

## References

[1] Espinosa N, Maurer MA (2015). Peroneal tendon dislocation. Eur J Trauma Emerg Surg.

[2] Malik AK, Mehta S, Solan M (2013). Significance of flake fracture with medial malleolar fracture. Foot Ankle Int..

[3] Kopp F (2008). Traumatic peroneal dislocation with medial malleolus fracture: a case report. Foot Ankle Int..

[4] Eckert WR, Davis EA (1976). Acute rupture of the peroneal retinaculum. J Bone Joint Surg Am..

[5] Herscovici D, Scaduto JM, Infante A (2007). Conservative treatment of isolated fractures of the medial malleolus. J Bone Joint Surg Br..

